# Targeting prohibitins induces apoptosis in acute myeloid leukemia cells

**DOI:** 10.18632/oncotarget.11333

**Published:** 2016-08-17

**Authors:** Helena Pomares, Claudia M Palmeri, Daniel Iglesias-Serret, Cristina Moncunill-Massaguer, José Saura-Esteller, Sonia Núñez-Vázquez, Enric Gamundi, Montserrat Arnan, Sara Preciado, Fernando Albericio, Rodolfo Lavilla, Gabriel Pons, Eva M González-Barca, Ana M Cosialls, Joan Gil

**Affiliations:** ^1^ Departament de Ciències Fisiològiques, Universitat de Barcelona-Institut d'Investigació Biomèdica de Bellvitge (IDIBELL), Barcelona, Spain; ^2^ Servei d'Hematologia, Institut Català d'Oncologia-IDIBELL, Barcelona, Spain; ^3^ CIBER-BBN, Networking Centre on Bioengineering, Biomaterials and Nanomedicine, Barcelona Science Park, Barcelona, Spain; ^4^ Department of Organic Chemistry, University of Barcelona, Barcelona, Spain; ^5^ School of Chemistry and Physics, University of KwaZulu-Natal, Durban, South Africa; ^6^ Laboratory of Organic Chemistry, Faculty of Pharmacy, University of Barcelona, Barcelona, Spain

**Keywords:** acute myeloid leukemia, apoptosis, prohibitins, BCL-2 family members, cancer

## Abstract

Fluorizoline is a new synthetic molecule that induces apoptosis by selectively targeting prohibitins (PHBs). In this study, the pro-apoptotic effect of fluorizoline was assessed in two cell lines and 21 primary samples from patients with debut of acute myeloid leukemia (AML). Fluorizoline induced apoptosis in AML cells at concentrations in the low micromolar range. All primary samples were sensitive to fluorizoline irrespectively of patients' clinical or genetic features. In addition, fluorizoline inhibited the clonogenic capacity and induced differentiation of AML cells. Fluorizoline increased the mRNA and protein levels of the pro-apoptotic BCL-2 family member NOXA both in cell lines and primary samples analyzed. These results suggest that targeting PHBs could be a new therapeutic strategy for AML.

## INTRODUCTION

Acute myeloid leukemia (AML) is a heterogeneous hematopoietic malignant disorder resulting from genetic alterations that lead to deregulation of proliferation, differentiation and cell death in hematopoietic progenitors [1]. Although complete remission is often achieved after high-intensity chemotherapy in younger patients with AML, 5-year overall survival rate is about 40%, since most patients become resistant to chemotherapy and frequently relapse. For these patients with relapsed/refractory disease therapeutic options are limited [1–3]. Accumulating evidence indicates that incomplete eradication of aberrant self-renewal and drug-refractory leukemia stem cells (LSC) in bone marrow (BM) niche sites is responsible for disease relapse [4]. Importantly, *TP53* alterations, that occur in around 8% of all AML patients and in 70% of patients with AML and complex karyotype, are associated to very bad prognosis [5, 6] and have a relevant role in the origin and evolution of therapy-related AML [7]. Therefore, new targets and innovative, more potent drugs are urgently needed to improve both clinical outcomes and long-term quality of life, especially for poor-risk patients and those not eligible for intensive treatment or BM transplantation.

Most chemotherapeutic drugs induce apoptosis of cancer cells. The execution of apoptosis depends on the balance between pro- and anti-apoptotic BCL-2 family members [8]. These proteins control the mitochondrial outer membrane permeabilization (MOMP) and the release of mitochondrial intermembrane proteins such as cytochrome *c* [9]. In AML, it has been proposed that mitochondrial apoptotic priming of myeloblasts provided by BH3-only profiling predicts chemotherapeutic success [10]. Furthermore, recent studies show the importance of mitochondria functionality in maintaining AML cells, thus emerging as potential drug target [11].

**Figure 1:**
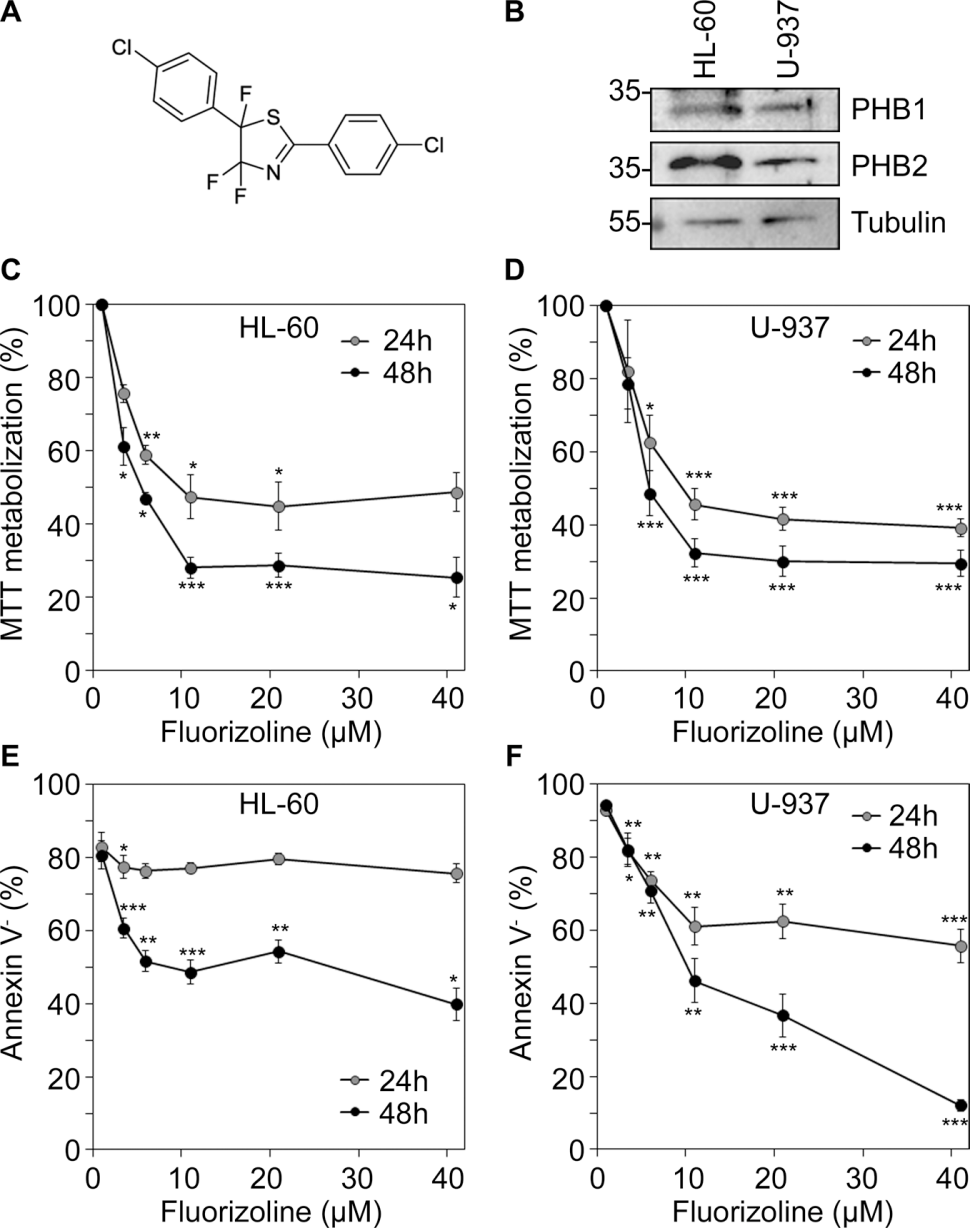
Cytotoxicity of fluorizoline in AML cell lines (**A**) Chemical structure of fluorizoline. (**B**) Whole cell lysates from HL-60 and U-937 cell lines were obtained and total protein levels of PHB1 and PHB2 were analyzed by western blot. Tubulin was used for loading normalization. (**C** and **E**) HL-60 cells and (**D** and **F**) U-937 cells were incubated for 24 and 48 h with increasing doses of fluorizoline ranging from 2.5 to 40 μM. (C and D) MTT metabolization was measured by absorbance and is expressed as the mean ± SEM (*n* ≥ 3) of the percentage of the value of untreated cells. (E and F) Viability was measured by analysis of phosphatidylserine exposure and is expressed as the mean ± SEM (*n* ≥ 3) of the percentage of non-apoptotic (annexin V negative) cells. Two-tailed paired Student's *t* test significant *p* values are indicated: *p < 0.05; **p < 0.01; ***p < 0.001 treated *versus* untreated cells.

Our group has described novel pro-apoptotic small molecules with fluorinated thiazole scaffolds [12]. The diaryl trifluorothiazoline compound 1a, hereafter referred to as fluorizoline (Figure 1A), was selected as the best apoptosis inductor in a wide range of cancer cell lines from different tissue origin, including hematopoietic cell lines, and with different p53 status, proving that fluorizoline exerts its anti-tumor action in a p53-independent manner [12]. Fluorizoline selectively binds to prohibitin (PHB) [12] and, strikingly, this protein is necessary for apoptosis induction by this compound [13]. Fluorizoline treatment induces mitochondrial-mediated apoptosis by controlling the expression of the BCL-2 family proteins. In this regard, induction of the pro-apoptotic BH3-only protein NOXA is required for fluorizoline-induced apoptosis and BIM is also involved depending on the cellular context [13].

Two homologous prohibitin proteins, PHB1 and PHB2, have been described [14, 15]. Increasing evidences link PHBs to tumorigenesis even though the role of PHBs in tumor growth and/or tumor suppression is still controversial [16, 17]. In acute promyelocytic leukemia cells (APL), PHBs co-immunoprecipitate with α-dystrobrevin [18]. Interestingly, nuclear PHB2 is an AKT substrate during all-*trans*-retinoic acid (ATRA)-induced differentiation and phosphorylation of PHB2 is necessary for cell survival [19]. On the other hand, it has been reported that downregulation of PHB expression protects from spontaneous apoptosis in ATRA-resistant cells [20]. The objective of this work was to investigate the effect of fluorizoline in AML cells and further dissect its mechanism of action.

## RESULTS

### Fluorizoline treatment induces apoptosis in AML cell lines

First, we tested the effect of fluorizoline (Figure 1A) in the AML cell lines HL-60 and U-937. Both cell lines express PHB1 and 2 (Figure 1B) and were sensitive to fluorizoline (Figure 1C–1F). When determining cell viability by the MTT assay, a dose-dependent decrease of cell viability was observed in both cell lines (Figure 1C–1D), with IC_50_ (half-maximal inhibitory concentration) values of 8 and 6 μM at 24 and 48 hours, respectively, in HL-60 cells, and 7 and 5 μM at 24 and 48 hours, respectively, in U-937 cells. Next, whether fluorizoline induced apoptosis in U-937 cells was analyzed by flow cytometry. Fluorizoline treatment induced the annexin V^+^/propidium iodide^−^ (PI^−^) and the annexin V^+^/PI^+^ cell populations (Supplementary Figure S1). Importantly, the pan-caspase inhibitor Q-VD-OPh was able to prevent the pro-apoptotic activity of fluorizoline and the annexin V^−^/PI^+^ population represented less than 6% of cells. Thus, we examined fluorizoline-induced apoptosis using annexin V staining by flow cytometry. Apoptotic cells were detected in the HL-60 cell line after 48 hours of fluorizoline treatment, reaching LD_50_ (half-maximal lethal dose) at 40 μM (Figure 1E). In U-937 cells, fluorizoline induced apoptosis in a dose-dependent manner (Figure 1F) with a mean LD_50_ value above 40 μM at 24 hours and 14 μM at 48 hours. Finally, the structurally similar inactive analog compound 2a [12] as well as equivalent concentrations of the vehicle DMSO did not affect cell viability (Supplementary Figure S2). Taken together, these data demonstrate that fluorizoline effectively induces apoptosis in AML cell lines.

### Fluorizoline treatment induces apoptosis in AML primary blasts

**Figure 2:**
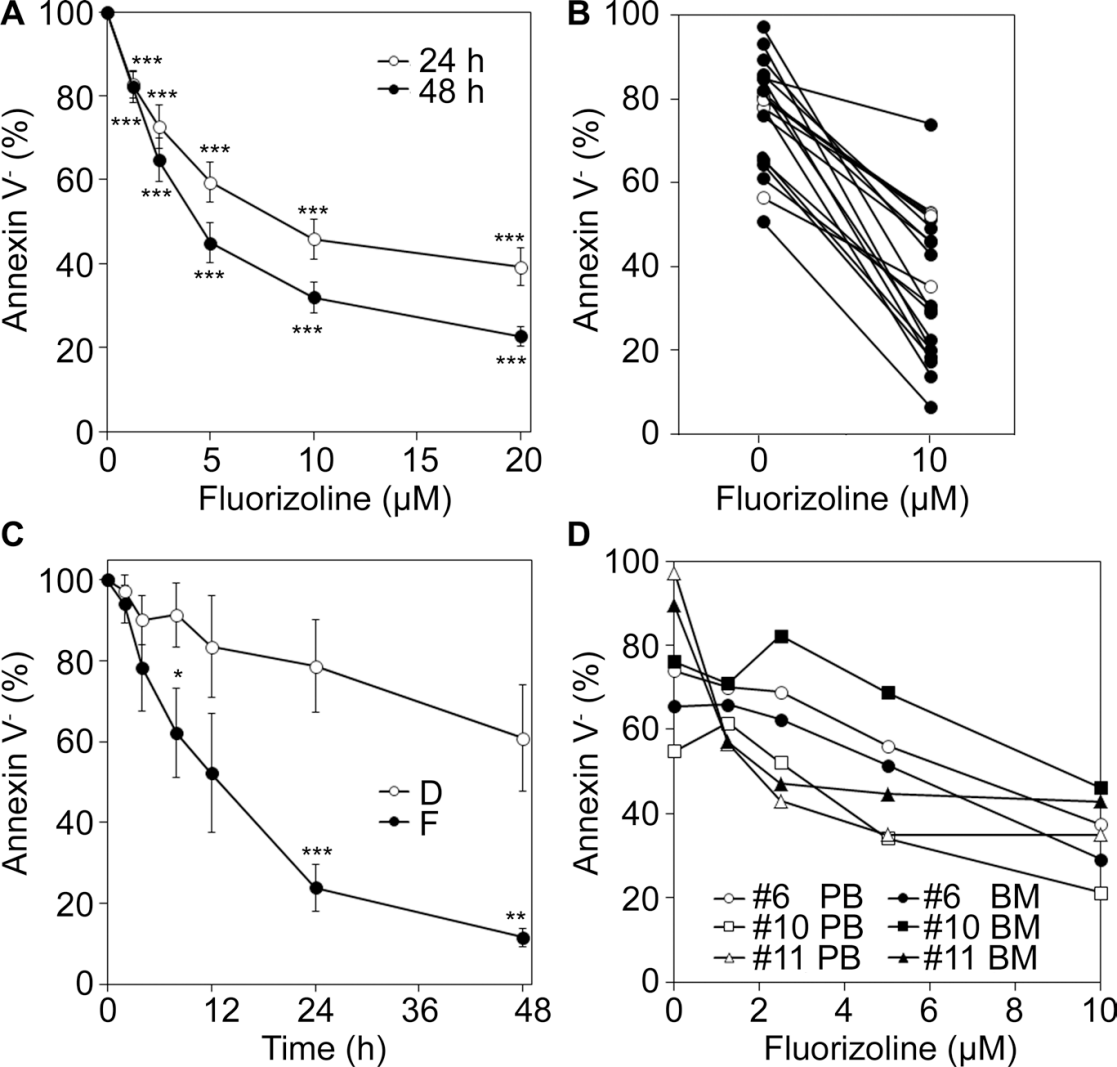
Cytotoxicity of fluorizoline in primary AML cells *ex vivo* (**A**) Dose response of fluorizoline on primary AML cells. BMMNC or PBMNC from 21 newly diagnosed AML patients were incubated for 24 and 48 h with increasing doses of fluorizoline ranging from 1.25 to 20 μM. (**B**) Blasts from 21 AML patients were incubated for 24 h without or with 10 μM fluorizoline. White filled shapes represent 5 patient samples with LD_50_ values >20 μM. (**C**) Time course of fluorizoline-induced apoptosis in AML cells. Cells from 6 patients (#13, 14, 16, 18, 20 and 21) were incubated for different times ranging from 2 to 48 h with 10 μM fluorizoline (F) (except patient #21, whose sample was treated with 2.5 μM fluorizoline) or with equivalent concentrations of the vehicle DMSO (D). (**D**) BMMNC or PBMNC from 3 patients (#6, 10 and 11) incubated for 24 h with increasing doses of fluorizoline ranging from 1.25 to 10 μM. (A, B, C and D) Viability was measured by analysis of phosphatidylserine exposure and is expressed as the percentage of non-apoptotic (annexin V negative) cells (in B and D) or as the percentage of the viability (annexin V negative) of untreated cells (in A) or as the percentage of the viability of cells at 0 h (in C). Data are shown as the mean ± SEM (in A and C). Two-tailed paired Student's *t* test significant *p* values are indicated: *p ≤ 0.05; **p ≤ 0.01; ***p ≤ 0.001 treated *versus* untreated (in A) or DMSO-treated cells at each time point (in C).

Next, the cytotoxicity of fluorizoline was evaluated in samples obtained from patients with AML who had not previously received any treatment (see Table 1 for details of patients and samples). PHBs protein levels were similar in all primary samples (data not shown and Figure 6). Cells from 21 different patients were exposed *ex vivo* to a range of fluorizoline concentrations (from 1.25 to 20 μM). The mutational status of *FLT3* and *NPM1*, the two most frequent mutations found in AML [3, 5], was analyzed in most patient samples. The association of the antileukemic effect of fluorizoline in AML primary samples according to cytogenetic risk categories or according to the clinical response to standard chemotherapy was analyzed by Fisher's exact test and no statistically significant associations were found (*p* > 0.5 in all categories) (Table 1). In leukemic primary BM or PB mononuclear cells (BMMNC and PBMNC, respectively) incubation with fluorizoline strongly reduced cell viability in a dose-dependent manner (Figure 2A). Most AML samples were sensitive to fluorizoline at 24 hours, and cell viability decreased from 75.8% ± 2.9% to 35.5% ± 4.0% after incubation with 10 μM fluorizoline (*n* = 20) (Figure 2B), with LD_50_ values ranging from 1.5 to 20 μM (median 8.0 ± 1.7 μM) for sensitive patient samples (*n* = 15) (Table 1 and Supplementary Figure S3). Longer exposition to fluorizoline for 48 hours slightly reduced the mean LD_50_ value to 5.3 ± 0.8 μM (*n* = 21) (Table 1), being all samples sensitive to the compound. It is noteworthy that no difference was observed between samples obtained at the debut and after disease progression, even those samples derived from the same patient, with median LD_50_ values of 10.3 ± 4.0 μM and 5.4 ± 1.6 μM at 24 and 48 hours, respectively (*n* = 6 samples at relapse; Supplementary Figure S4). Incubation with 10 μM fluorizoline induced a time-dependent decrease of cell viability being evident at 8 hours in a subset of patient samples (Figure 2C). Blasts from PB and BM derived from the same patient were similarly sensitive to fluorizoline (Figure 2D). These results therefore indicate that fluorizoline induces apoptosis in primary AML cells.

**Table 1:** Patients' characteristics

Patient No.	Age/Gender	WHO subtype	FAB	Cytogenetics	*FLT3/NPM1*	Risk^Δ^	Clinical response^•^	24 h^†^	48 h^†^
1	65/M	AML with myelodysplasia-related changes	M0	47,XY,t(2;11)(p21;q23),-9,-14,+22,+mar1,+mar2[16]/46,XY[4]	wt/wt	H	CR	> 5	2.5
2	80/F	AML with myelodysplasia-related changes^*^	M0	46,XX,t(10;20)(q22;q11.2)[19]/46,XX[1]	nd/nd	ND	Ut	6	4
3	75/F	AML with myelodysplasia-related changes	M1	53-54,X,-X,+4,+6,+8,+9,+12,+14,+21,+21 [4],+mar[cp20]	wt/wt	H	Ut	20	5
4	64/M	AML without maturation	M1	47,XY,+8[18]/46,XY[2]	wt/wt	I	R	15	5
5	86/F	AML with myelodysplasia-related changes^**^	ND	ND	nd	ND	Ut	3	3
6	34/F	AML with t(9;11)(p22;q23); MLLT3-MLL	M5a	46,XX,t(9;11)(p22;q23)[20]	wt/wt	H	CR	9	3
7	40/M	AML with t(3;3)(q21;q26.2); RPN1-EVI1	M7	45,X,-Y,t(3;3)(q21;q26)[18]/46,XY[2]	wt/wt	H	R	> 20	12
8	79/M	Acute undifferentiated leukemia with ambigous lineage	M0	46,XY[20]	nd	ND	U	5	4.5
9	57/M	AML with mutated NPM1	M5b	46,XY[30]	wt/mut	L	CR	> 20	4
10	66/F	AML with myelodysplasia-related changes and mutated NPM1	M2	46,XX[30]	wt/mut	L	CR	13	8
11	57/M	AML with myelodysplasia-related changes^*^	M1	47,XY,+8[20]	itd/wt	H	CR	5	3
12	55/M	AML with mutated NPM1	RAEB-T	46,XY[20]	wt/mut	L	CR	2.5	2
13	55/F	AML with inv(16)(p13.1q22); CBFB-MYH11^***^	M4Eo	46,XX[20]	wt/ wt	L	CR	11	6
14	55/M	AML with mutated NPM1	M5a	47,XY,+8[13]/46,XY[7]	itd/mut	H	ND	4	4
15	62/M	AML with myelodysplasia-related changes	M2	46,XY,del(5)(q13q33)[10]/46,XY[10]	wt/wt	H	R	20	7.5
16	65/F	AML with mutated NPM1	M1	46,XX[18]	itd/ mut	L	U	> 20	8
17	24/M	AML with myelodysplasia-related changes	M1	47,XY,+8[11]/52,sl,+3,+6,+10,+13,+19[9]	itd/ wt	H	R	> 20	5
18	74/M	AML with myelodysplasia-related changes	RAEB-T	46,XY,-7,+9[12]/46,XY[5]	wt/wt	H	U	2.5	2.5
19	60/M	AML with maturation	M2	46,XY[8]	wt/wt	I	CR	3	2
20	40/M	AML without maturation	M1	46,XY[30]	wt/wt	I	CR	> 20	17
21	56/F	Acute promyelocytic leukemia with t(15;17)(q22;q12); PML-RARA	M3	46,XX,t(15;17)(q24;q21)[20]	nd/nd	L	CR	1,5	3

Patient samples were considered sensitive when a LD50 < 20 μM fluorizoline was reached.

† LD50 values for sensitive patient samples at 24 and 48 hours are shown.

Δ Cytogenetic and molecular risk categories defined by the Medical Research Council and the European LeukemiaNet prognostic classification.

• Idarubicin at dose of 12 mg/m^2^ daily for 3 days and cytarabine at a dose of 200 mg/m^2^ daily for 7 days as a continuous infusion were considered the standard induction therapy. The consolidation treatment consisted of high dose of cytarabine or allogeneic hematopoietic stem cell transplantation depending on the AML risk classification according to the CETLAM protocol.

* Morphologic criteria.

** Multilineage dysplasia and previous history of myelodysplastic syndrome.

*** Cryptic translocation. CBFB-MYH11 was demonstrated by molecular genetic studies. FAB, French–American–British classification systems; M, male; F, female; RAEB-T, refractory anemia with excess of blasts in transformation; WT, wild type; D835, mutation of the Asp codon 835 within the activation loop of the FLT3 gene; Mut, mutated; ND, not determined; H, high; I, intermediated; L, low; CR, complete remission; U, untreated; R, refractory; CETLAM, Grupo Cooperativo de Estudio y Tratamiento de las Leucemias Agudas y Mielodisplásicas.

**Figure 3:**
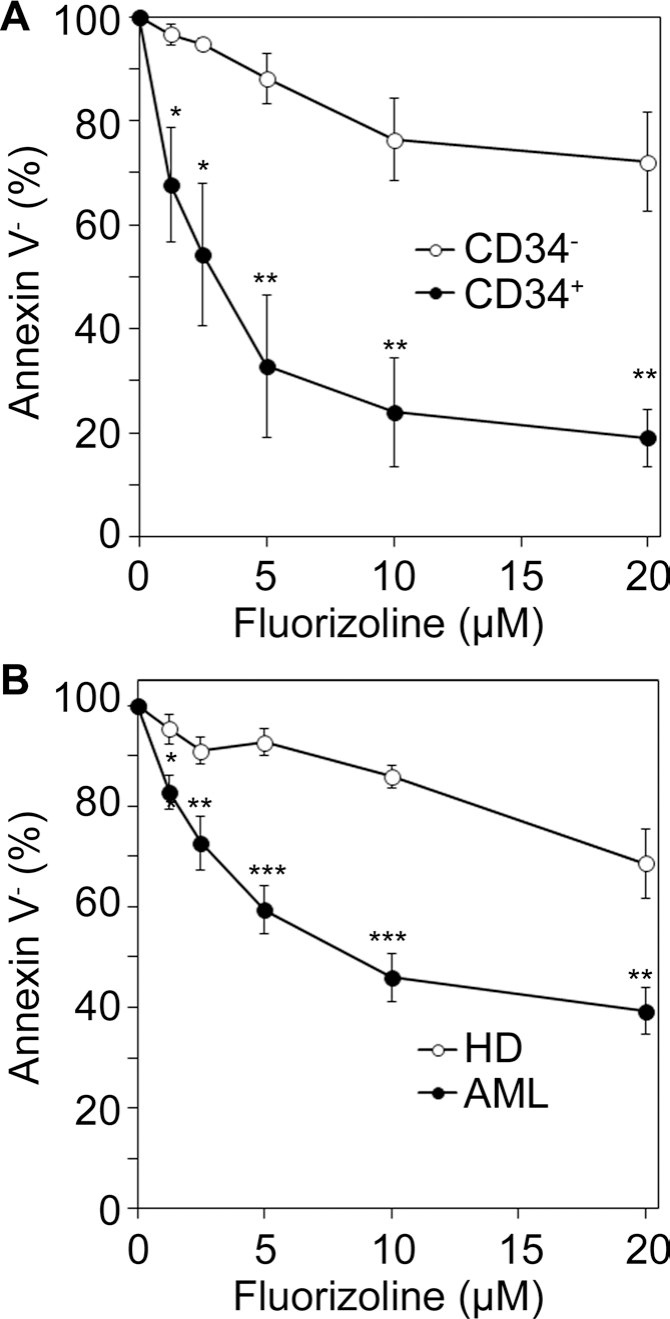
*Ex vivo* cytotoxicity of fluorizoline in AML cells and in healthy bone marrow and blood myeloid cells (**A**) Dose response of the cytotoxic effect of fluorizoline on CD34^+^ and CD34^−^ populations. BMMNC or PBMNC from 6 AML patients (#1, 2, 18, 19, 20 and 21) were incubated for 48 h with increasing doses of fluorizoline ranging from 1.25 to 20 μM. Viability was measured on CD34^−^ and CD34^+^ populations. (**B**) Comparative dose response of the cytotoxic effect of fluorizoline on normal hematologic cells and AML cells. BMMNC from 8 healthy BM or PBSC from one G-CSF-mobilized healthy donor (HD), and BMMNC or PBMNC from 21 newly diagnosed AML patients (AML) were incubated for 24 h with increasing doses of fluorizoline ranging from 1.25 to 20 μM. In normal cells viability was measured on total buffy coat population. (A and B) Viability was measured by analysis of phosphatidylserine exposure and is expressed as the percentage of the viability (annexin V negative) of untreated cells. Data are shown as the mean ± SEM. Two-tailed unpaired Student's *t* test significant *p* values are indicated: *p ≤ 0.05; **p ≤ 0.01; ***p ≤ 0.001 CD34^+^ *versus* CD34^−^ cells, or normal *versus* malignant cells.

To examine the effects on the leukemic cells more specifically, apoptosis induction was assayed in the CD34^+^ and CD34^−^ populations of six AML specimens. The reduction in cell viability in the presence of fluorizoline was higher within the CD34^+^ population (LD_50_ value of 4.5 ± 2.3 μM at 48 hours) compared to the CD34^−^ population, which was more resistant (LD_50_ value higher than 20 μM at 48 hours) (Figure 3A). This suggests that the fluorizoline cytotoxic effect is preferentially displayed on the AML compartment.

Finally, to evaluate the cytotoxicity of fluorizoline in non-malignant cells, the effect of fluorizoline on normal BMMNC and normal hematopoietic stem cells (PBSC) was assessed (see Table 2 for details of healthy volunteers and samples). Incubation with increasing doses of fluorizoline slightly reduced the percentage of viable normal cells (68.6% ± 7.0% of viable cells after 24 hours of treatment with 20 μM fluorizoline) (Figure 3B) with a median LD_50_ value above 20 μM both at 24 and 48 hours (Table 2), suggesting a higher resistance of normal hematopoietic and stromal cells to fluorizoline, as compared to leukemic cells.

**Table 2:** Healthy donors' characteristics

Donor No.	Disease^*^	Sample origin	LD_50_(μM) 24 h^*^	LD_50_(μM) 48 h^*^
1	DLBCL	BM	> 20	> 20
2	HD/G-CSF	Apheresis	> 20	> 20
3	HD	BM	> 20	> 20
4	Adenopathy	BM	> 20	> 20
5	FL	BM	> 20	> 20
6	Trombocytosis	BM	> 20	> 20
7	FL	BM	> 20	13
8	DLBCL	BM	> 20	> 20
9	FL	BM	16	8

Donors were considered sensitive when a LD_50_ < 20 μM fluorizoline was reached.

* LD_50_ values for sensitive donors are shown. DLBCL, diffuse large B-cell lymphoma; HD, healthy donor; FL, follicular lymphoma.

### Fluorizoline inhibits the clonogenic capacity and induces differentiation of AML cells

We performed methylcellulose colony-forming unit (CFU) assays to determine whether fluorizoline would functionally affect the ability of cells to form leukemic cell colonies. CFU assays showed that fluorizoline significantly reduces clonogenic capacity of U-937 cells at 5 μM and completely abolishes it at 10 μM (mean CFU respect to untreated cells, 49.9 ± 5 % for 5 μM and 1.9 ± 1% for 10 μM; *n* = 4). Next, we assessed the effects of fluorizoline on human primary leukemia blasts isolated from 5 AML patients. Fluorizoline treatment strongly reduced the ability of primary AML cells to form colonies (mean CFU respect to untreated cells, 39.9 ± 5 % for 2.5 μM and 5.3 ± 1.5 % for 10 μM; *n* = 5).

**Figure 4:**
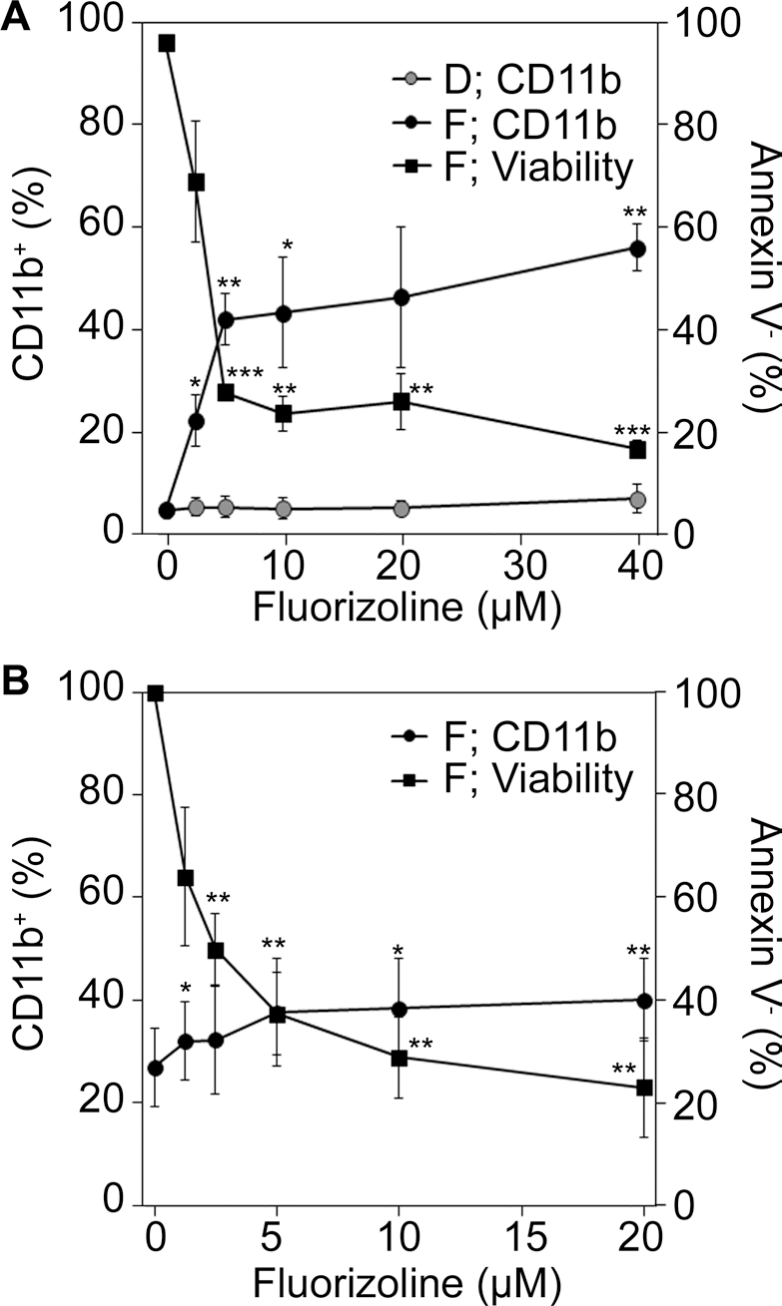
Effect of fluorizoline on the expression of the differentiation marker CD11b in AML cells (**A**) U-937 cells were incubated for 48 h with increasing doses of fluorizoline (F) ranging from 2.5 to 40 μM or equivalent concentrations of the vehicle DMSO (D), and CD11b expression and viability were analyzed. (**B**) Blasts from AML patients (#6, 17, 18 and 22) were incubated for 48 h with increasing doses of fluorizoline (F) ranging from 1.25 to 20 μM, and CD11b expression and viability were analyzed. (A and B) CD11b expression was measured by flow cytometry and is expressed as the percentage of CD11b^+^ population. Viability was measured by analysis of phosphatidylserine exposure and is expressed as (A) the percentage of non-apoptotic (annexin V negative) cells and (B) the percentage of the viability (annexin V negative) of untreated cells. Data are shown as the mean ± SEM of (A) 3 independent experiments and (B) at least 2 AML patients (*n* = 2 at 24 h; *n* = 4 at 48 h). Patient #22 sample does not appear in Table 1 as viability data are not available at 24 h. Two-tailed paired Student's *t* test significant *p* values are indicated: *p < 0.05; **p < 0.01; ***p < 0.001 treated *versus* untreated cells.

We next assessed the effect of fluorizoline on myeloid maturation of U-937 cells as well as AML patient-derived cells. The expression of the granulocyte/monocyte/macrophage differentiation marker CD11b [19, 21] was upregulated at 24 hours of incubation with fluorizoline, being especially evident after 48 hours of treatment (not shown). The induction of CD11b surface marker expression by fluorizoline was dose-dependent (12.1- and 1.5-fold induction in 40 μM fluorizoline-treated U-937 cells (Figure 4A) and 20 μM fluorizoline-treated *de novo* AML primary samples (Figure 4B), respectively, compared to DMSO-treated cells at 48 hours) and occurred in parallel to cell viability decrease (Figure 4A and 4B). Conversely, the vehicle DMSO did not induce the expression of CD11b (Figure 4A and Supplementary Figure S5). These findings suggest that fluorizoline acts as a differentiation-inducing drug of AML cells.

### Fluorizoline induces upregulation of NOXA in AML cell lines

**Figure 5:**
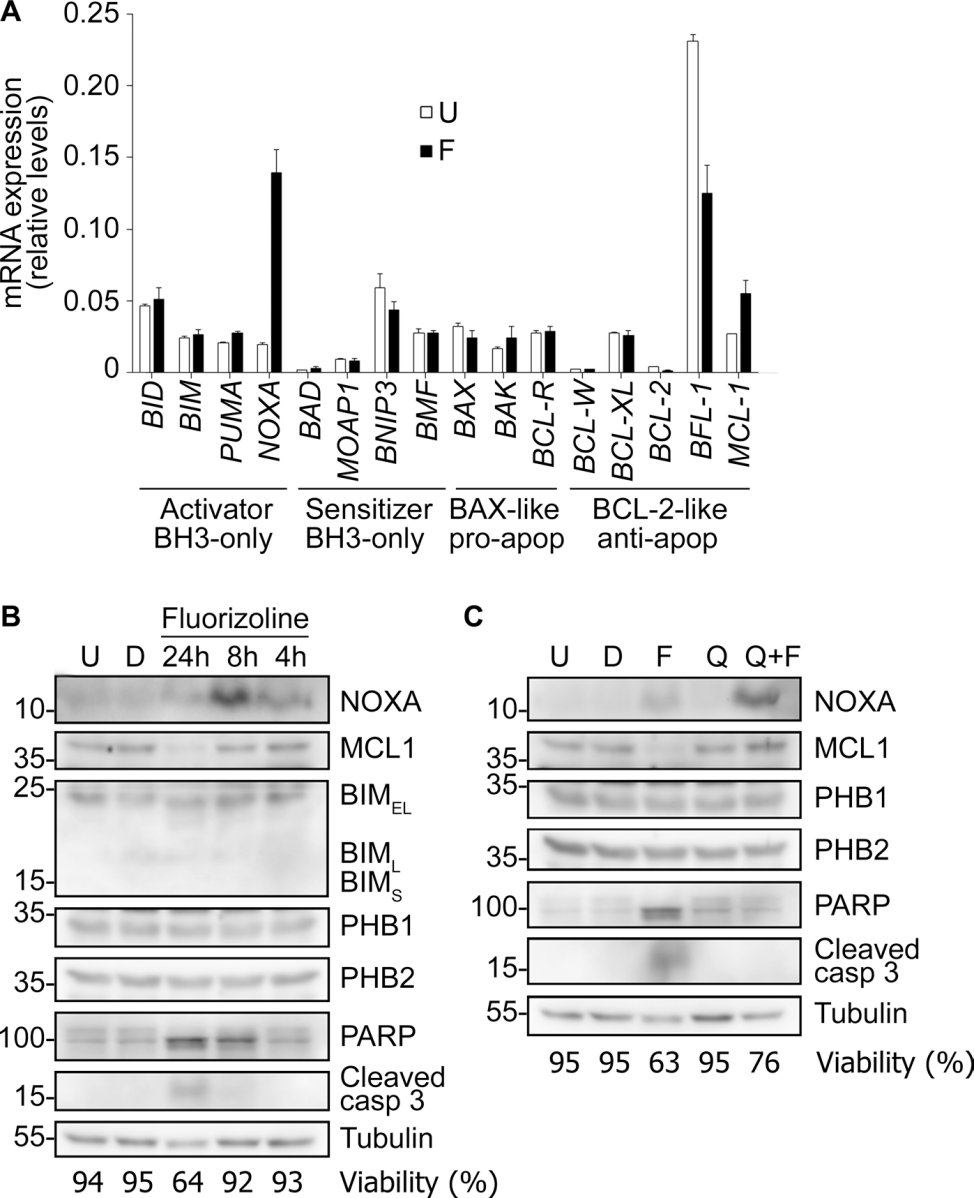
Fluorizoline modulates the expression of BCL-2 family members in the U-937 AML cell line (**A**) U-937 cells were untreated (U) or treated with 10 μM fluorizoline (F) for 8 h. BCL-2 family members mRNA levels were analyzed by RT-MLPA. Data show the mean ± SEM (*n* = 2) of the mRNA expression levels. (**B**) U-937 cells were untreated (U) or treated with equivalent concentrations of the vehicle DMSO (D) for 24 h and with 5 μM fluorizoline for 4, 8 and 24 h, as indicated. (**C**) U-937 cells were untreated (U) or pre-incubated with 20 μM caspase inhibitor Q-VD-OPh (Q) for 30 min and then treated with 5 μM fluorizoline (F) for 24 h. Cells were also incubated with equivalent concentrations of the vehicle DMSO (D) for 24 h. (B and C) Protein levels from whole cell lysates were analyzed by western blot. Tubulin was used as a loading control. These are representative images of at least three independent experiments. Viability was measured by analysis of phosphatidylserine exposure and is expressed as the percentage of non-apoptotic (annexin V negative) cells.

To gain insight into the molecular mechanism of fluorizoline-induced cytotoxicity in AML, changes in overall apoptosis-related gene expression profile were analyzed by reverse transcriptase multiplex ligation-dependent probe amplification (RT-MLPA) in the U-937 cell line. Fluorizoline mostly upregulated the BH3-only *NOXA* at the mRNA level from the early 4 hours of incubation (3.5-, 6.6- and 3.1-fold induction at 4, 8 and 24 hours of incubation, respectively) (Figure 5A and Supplementary Figure S6A). Also, fluorizoline induced the expression of NOXA protein from the early 4 hours and the degradation of the anti-apoptotic MCL-1 after 24 hours of incubation (Figure 5B). Both NOXA mRNA and protein highest expression levels were observed after 8 hours of incubation, and the expression was higher than in the untreated conditions until 24 hours (Supplementary Figure S7). Neither BIM nor PHB1/2 protein levels were modified upon fluorizoline treatment in U-937 cells. It should be noted that BIM_EL_ was the main expressed BIM isoform in U-937 cells [22, 23] (Figure 5B). Regarding the apoptotic features involved in the cytotoxic effect of fluorizoline, the cleaved fragment of caspase 3 as well as the proteolysis of the caspase substrate PARP were found at 8 hours of incubation with fluorizoline, and both events were clearly detected after 24 hours of treatment (Figure 5B). Conversely, the inactive compound 2a did not induce caspase 3 and PARP cleavage nor NOXA upregulation (Supplementary Figure S6A and S6B). Interestingly, fluorizoline-induced NOXA upregulation was still detected whereas MCL-1 degradation was reverted when caspase activity was blocked with the pan-caspase inhibitor Q-VD-OPh (Figure 5C and Supplementary Figure S7B), thus demonstrating that only the induction of NOXA precedes caspase activation. These results indicate that fluorizoline causes an increase of NOXA mRNA and protein levels prior to caspase activation and these modulations could explain the apoptotic outcome observed in the AML cell line U-937.

**Figure 6:**
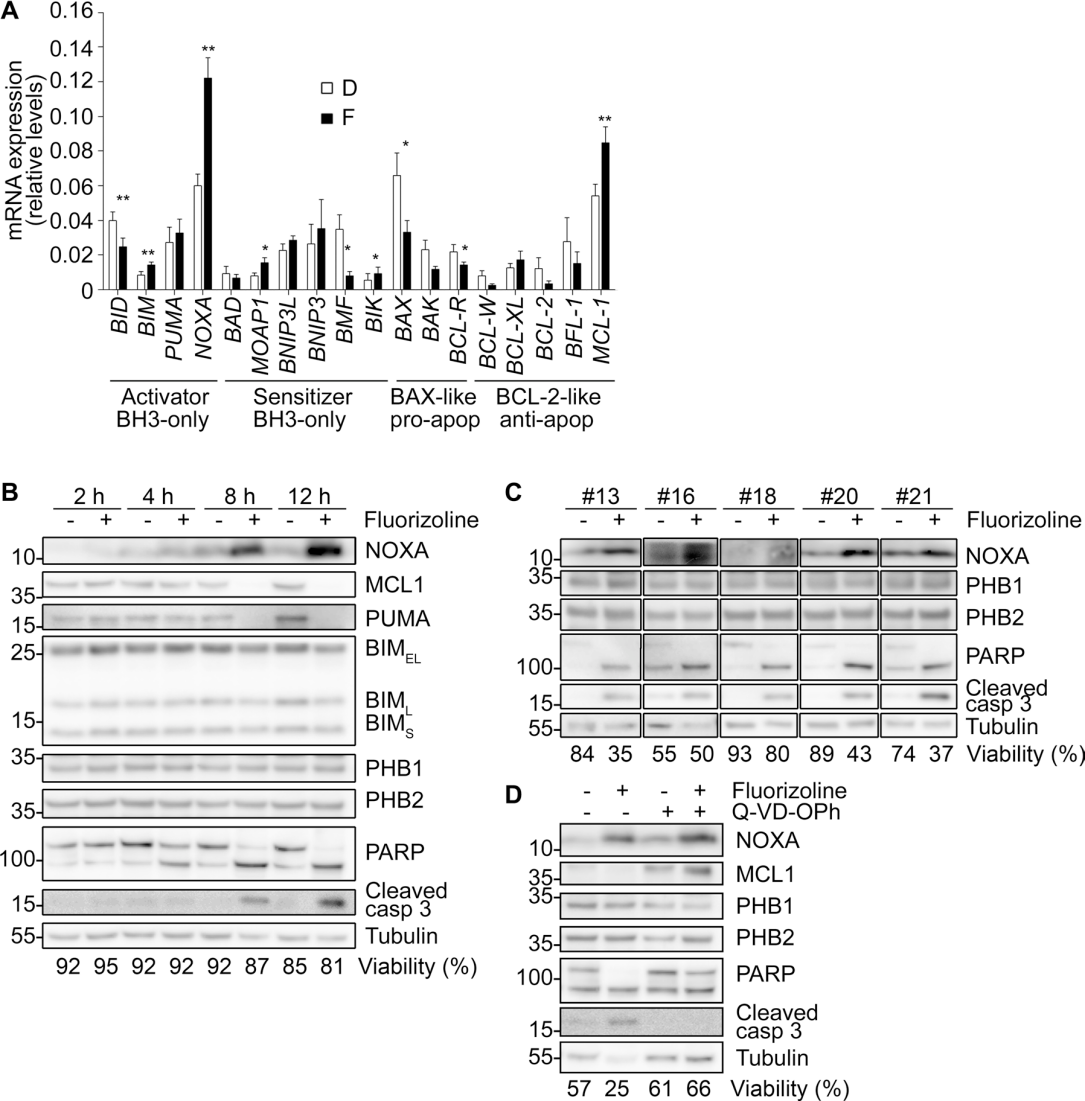
Induction of NOXA mRNA and protein levels upon fluorizoline treatment in primary AML samples (**A**, **B** and **C**) BMMNC or PBMNC from patients #13, 14, 16, 18, 20 and 21 were incubated for different times ranging from 2 to 24 h with 10 μM fluorizoline (F or +) (except patient #21, whose sample was treated with 2.5 μM fluorizoline) or with equivalent concentrations of the vehicle DMSO (D or -). (A) After 8 h of treatment, RNA from cells was extracted and analyzed by RT-MLPA and the results for BCL-2 family members are shown as the mean ± SEM (*n* = 6) of the mRNA expression levels. Two-tailed paired Student's *t* test significant *p* values are indicated: *p ≤ 0.05, **p ≤ 0.01, ***p ≤ 0.001 treated *versus* untreated cells. (B) After the times stated at the figure, cells from patient #14 were collected. (C) After 8 h of treatment, cells from patients #13, 16, 18, 20 and 21 were collected. (**D**) Cells from patient #20 were pre-incubated with 20 μM caspase inhibitor Q-VD-OPh for 30 min and then treated with 10 μM fluorizoline for 24 h. (B, C and D) Cells were lysed and analyzed by western blot. Tubulin was used for loading normalization. Viability was measured by analysis of phosphatidylserine exposure and is expressed as the percentage of non-apoptotic (annexin V negative) cells. These are representative patient samples of at least three analyzed (*n* = 6 for B and C; *n* = 3 for D).

### NOXA is upregulated by fluorizoline in primary AML cells

To validate the mechanism of apoptosis induction upon fluorizoline treatment in primary AML samples, the changes in apoptosis-related gene expression were analyzed. Treatment of patient samples with fluorizoline increased the expression of the pro-apoptotic BCL- 2 family members *BIM*, *NOXA* and *MOAP1* in a time-dependent manner, along with a decrease in the mRNA levels of the anti-apoptotic members *BCL-W*, *BCL-2* and *BFL-1*, although these downregulations were not statistically significant (Figure 6A and Supplementary Figure S8). The induction timing of these genes was patient-dependent and it mostly occurred during the first 8 hours of treatment, preceding the loss of cell viability in some cases (Supplementary Figure S9). At longer times, we also observed the induction of *PUMA* mRNA levels (Supplementary Figure S8). The expression of other apoptosis-related genes was also regulated upon fluorizoline treatment: *p21* was upregulated and some members of the IAP family (*HIAP2*, *SURVIVIN* and *NAIP*) were downregulated (Supplementary Figure S8). Modulation of the mRNA levels of *NOXA* led to upregulation of the protein (Figure 6B and 6C). The increment of NOXA protein expression was time-dependent and it was detected at the early 4 hours of incubation when cell viability was not affected yet (Figure 6B and Supplementary Figure S7). NOXA accumulation also preceded downregulation of anti-apoptotic MCL-1. PUMA protein expression decreased or remained stable, and BIM and PHBs protein levels were not modified upon fluorizoline treatment (Figure 6B and 6C). Similarly, expression levels of PHBs were not modified in normal hematopoietic cells (Supplementary Figure S10). Also, fluorizoline clearly induced caspase 3 cleavage and PARP proteolysis after 8 hours of treatment (Figure 6B and 6C), thus confirming an apoptotic mechanism. Finally, as described for the U-937 AML cell line, increases in NOXA preceded caspase activation, as pre-incubation with the pan-caspase inhibitor Q-VD-OPh did not block its upregulation (Figure 6D and Supplementary Figure S7). In contrast, caspase inhibition abolished the fluorizoline-mediated decrease in MCL-1, thereby demonstrating that only NOXA upregulation is a caspase-independent event in primary AML samples. Taken together, these results indicate that NOXA is involved in the mechanism of fluorizoline-induced apoptosis in primary AML cells.

## DISCUSSION

In the present study we describe that the prohibiting-binding compound fluorizoline induces apoptosis in AML cells, including a series of primary samples. Our results suggest that PHB has a pro-survival role in AML that is inhibited by the selective binding of fluorizoline. Furthermore, we describe that flourizoline is able to impair the ability of primary AML cells to form colonies, which provides evidence that fluorizoline inhibits the growth of AML progenitors cells.

Treatment with ATRA plus arsenic trioxide (ATO), which degrades the PML/RARα fusion protein, results in the differentiation of APL cells and is the only highly effective molecular targeted strategy for AML [24]. Interestingly, we have observed that fluorizoline induces differentiation of primary AML cells even though these patients did not express the translocation t(15;17) and could not benefit from ATRA differentiating therapy. Thus, the mechanism by which fluorizoline induces differentiation of AML cells is independent of the expression of the PML/RARα fusion protein.

The mitochondrial or intrinsic pathway of apoptosis, controlled by the BCL-2 family proteins, is thought to be the main mechanism of AML cell killing by chemotherapy [26]. Increased levels of the anti-apoptotic family members BCL-2, BCL-X_L_ and/or MCL-1 have been found to predict poor prognosis of AML patients to chemotherapy [27–30]. Expression of BCL-X_L_, BCL-2 and MCL-1 is heterogeneous and overlapping within AML subtypes, suggesting functional redundancy [31]. Molecular studies revealed that MCL-1 is overexpressed in AML and plays an even more important role than BCL-2 or BCL-X_L_ in protecting AML cells from apoptosis [32, 33]. AML bulk and stem cells are dependent on the anti-apoptotic BCL-2 members for survival, and their inhibition causes cell death in AML cells [34, 35].

Expression analysis revealed a consistent upregulation of NOXA both in cell lines and primary AML cells upon fluorizoline treatment. Fluorizoline induced increases in NOXA protein levels prior to caspase activation, which could explain the apoptotic outcome in AML cells. The expression of BIM protein was not modulated by fluorizoline in AML cells, similarly to other cells of hematological origin [13]. Fluorizoline also downregulated the expression of MCL-1 in AML cells, although this effect was dependent on caspase activation and would not be involved in the primary apoptotic mechanism of the drug. NOXA has been described as a particularly relevant pro-apoptotic BCL-2 family member in different cell types [36]. NOXA protein is induced in AML cells by HDAC inhibitors [37, 38], MDM2 inhibition [39], Aurora B kinase inhibitors [40], inhibition of Nedd8 activating enzyme [41, 42], hyperforin [43] and dihydroartemisinin [44]. Our results emphasize the role of NOXA in the induction of apoptosis in AML cells.

Novel therapies that effectively achieve cancer cell killing with minimal toxicity to normal cells are urgently needed for AML. Our results indicate that fluorizoline induces apoptosis in AML cells through NOXA upregulation by preferentially affecting leukemic immature progenitor cells while sparing normal cells. This data suggest that targeting PHBs could be a new and interesting therapy for AML.

## MATERIALS AND METHODS

### Cell lines

Human AML cell lines HL-60 and U-937 (kindly provided by Dr. Pablo Engel, Universitat de Barcelona) were maintained in RPMI 1640 culture medium supplemented with 10% heat-inactivated fetal bovine serum (FBS), 2 mM L-glutamine, 100 U/mL penicillin, and 100 μg/mL streptomycin (all supplied by Biological Industries, Kibbutz Beit HaEmek, Israel) at 37°C in a humidified atmosphere containing 5% carbon dioxide.

### Primary AML samples, healthy donors and cell isolation

PB samples and BM aspirates from 21 newly diagnosed and untreated patients with AML (Table 1) were obtained after informed consent in accordance with protocols approved by the Human Research Ethics Committees of the Hospital ICO-Duran i Reynals, L'Hospitalet de Llobregat, Barcelona, Spain. AML was diagnosed and classified according to standard clinical and laboratory criteria, according to the classification of the World Health Organization (WHO) [45]. Caryotype was determined by G-banding [46]. Internal tandem duplications (ITD) and mutations of the gene *FLT3* (*fms related tyrosine kinase 3*) were determined by polymerase chain reaction (PCR) [47, 48]. Mutations of the gene *NPM1* (*nucleophosmin*) were determined by PCR [49].

PBMNC and BMMNC were isolated by centrifugation on a Biocoll (Biochrom AG, Berlin, Germany) gradient and cryopreserved in liquid nitrogen in the presence of 10% dimethyl sulfoxide (DMSO) (Sigma-Aldrich Inc, St Louis, MO, USA). MNC were cultured immediately after thawing or isolation at a concentration of 1 × 10^6^ cells/mL in RPMI 1640 culture medium supplemented with 10% heat-inactivated FBS, 2 mM L-glutamine, 100 U/mL penicillin, and 100 μg/mL streptomycin (all from Biological Industries) at 37°C in a humidified atmosphere containing 5% carbon dioxide.

Normal BMMNC was obtained from apheresis-collected granulocyte colony-stimulating factor (G-CSF)-mobilized progenitor-cell from PB of 3 healthy donors were used as a source of normal CD34^+^ cells.

Circulating B and T lymphocytes from healthy donors were purified by negative selection (RosetteSep™ Human B Cell Enrichment Cocktail and RosetteSep™ Human T Cell Enrichment Cocktail, StemCell Technologies, respectively) previously to Biocoll gradient centrifugation and then used as normal hematopoietic cells.

### Reagents

Fluorizoline (a diaryl trifluorothiazoline; see molecular structure in Figure 1A) and compound 2a (a non-fluorinated diaryl thiazole synthetic precursor of fluorizoline; see molecular structure in Supplementary Figure 2A) were synthesized as previously described [12] and dissolved in DMSO at 20 or 100 mM. Allophycocyanin (APC)-Cy7-labeled antibody against human CD11b was purchased from BD Biosciences (Franklin Lakes, NJ, USA). APC-Alexa Fluor 750 anti-human CD33 was purchased from Beckman Coulter (Marseille, France). APC-Cy7 anti-human CD34 was from Sony Biotechnology Inc. (Champaing, IL, USA). APC-conjugated annexin V was purchased from eBiosciences (San Diego, CA, USA). Q-VD-OPh was from R&D Systems (Minneapolis, MN, USA). MTT ((3-4,5-dimethyl-2-thiazolyl)-2,5-diphenyl-2H-tetrazolium bromide) was from Sigma-Aldrich. PI was purchased from eBiosciences (San Diego, CA, USA).

### Analysis of cell viability by the MTT assay

HL-60 and U-937 cells (1.25 × 10^5^ cells/well) were incubated in the absence or in the presence of the indicated factors, in a final volume of 250 μL. After 24 or 48 hours, 25 μL MTT reagent (prepared at 5 mg/mL in phosphate-buffered saline (PBS)) was added to each well for an additional 3 hours. The blue MTT formazan precipitate was dissolved in 250 μL isopropanol:1 M HCl (24:1). Then, 100 μL of this cell lysate were plated in a 96-well plate in triplicate and the absorbance values at 570 nm were measured on a multiwell plate reader. The half-maximal inhibitory concentration (IC_50_) was defined as the concentration of drug required to reduce the MTT metabolization ability by 50%.

### Analysis of cell purity and cell viability by flow cytometry

Cell viability was assessed by phosphatidylserine exposure and measured as the percentage of annexin V-negative cell population. After incubation of 1.25 × 10^5^ cells with the indicated factors and times, cells were then washed in annexin-binding buffer (ABB) and incubated in 100 μL ABB with 0.5 μL annexin V-APC for 10 min in the dark and then diluted with ABB to a final volume of 150 μL. Occasionally, 3.75 μL PI were added after annexin V staining and immediately before acquisition to measure the percentage of annexin V^−^/PI^−^ live cells. In AML samples with infiltration lower than 80% blasts, double staining with anti-CD33 or anti-CD34, and annexin V was performed. In brief, cells were washed in ABB and incubated in 50 μL ABB containing 0.5 μL anti-CD33 or anti-CD34 for 10 min in the dark and thereafter stained with annexin V, as previously mentioned. Cells were acquired using the FACSCanto^TM^ II flow cytometer (Becton Dickinson, Franklin Lakes, NJ, USA) and data of bulk leukemic/normal and CD33^+^- or CD34^+^-gated leukemic/normal progenitor cells were analyzed using the FACSDiva^TM^ software (Becton Dickinson). Half-maximal lethal dose (LD_50_) was defined as the concentration of drug required to reduce the cell viability by 50%. Patient samples were considered as “sensitive” to fluorizoline when cell viability was lower than 80% after incubation with 20 μM during 48 hours.

### Colony-forming unit (CFU) assay

The analysis of the clonogenic capacity after exposition to the fluorizoline was performed using a methylcellulose-based medium with recombinant cytokines and erythropoietin (EPO) for human hematopoietic progenitor cells (MethoCult^TM^ H4034 Optimum, StemCell Technologies). U-937 cells and primary AML cells were pretreated for 24 hours with the indicated factors, as previously mentioned. Then, cells (1 × 10^3^ U-937 cells or 50 × 10^3^ primary AML cells) were diluted in 100 μL serum-free IMDM with 25 mM HEPES, homogeneously mixed with 1 mL MethoCult^TM^ medium, seeded in duplicate cultures and maintained at 37°C with 5% CO_2_ in air in a humidified atmosphere. Colonies (CFU) were screened based on morphology and cellularity at day 10 (U-937 cells) or day 14 (primary AML cells) using an inverted microscope. A colony was defined as a cluster of > 40 cells.

### Differentiation assay

Myeloid maturation induction was assessed by cell surface marker expression analysis [19, 21]. U-937 and primary AML cells were treated as indicated for cell viability assays. After 24 and 48 hours of incubation cells were collected and stained in 50 μL PBS buffer containing 2 mM EDTA and 0.5% bovine serum albumin (BSA) with 0.5 μL anti-human CD11b for 10 min in the dark and then diluted with buffer to a final volume of 150 μL. In parallel, cell viability was analyzed by annexin V staining in separated tubes. Surface expression of the antigen was analyzed by flow cytometry. In these experiments vehicle-treated cells were also included in order to discard DMSO-induced myeloid differentiation [19].

### Reverse transcriptase multiplex ligation-dependent probe amplification (RT-MLPA)

RNA was isolated from cultured 3–5 × 10^6^ cells by the RNeasy Micro kit (Qiagen GmbH, Hilden, Germany) according to the manufacturer's protocol. RNA was analyzed by RT-MLPA using SALSA MLPA KIT R011-C1 Apoptosis mRNA from MRC-Holland (Amsterdam, The Netherlands) for the simultaneous detection of 40 messenger RNA molecules, including apoptosis-related genes [13, 50]. In brief, RNA samples (200 ng total RNA) were first reverse transcribed using a gene-specific primer mix. The resulting cDNA was annealed overnight at 60°C to the RT-MLPA probe mix. Annealed oligonucleotides were ligated by adding Ligase-65 (MRC-Holland) and incubated at 54°C for 15 min. Ligation products were amplified by PCR (35 cycles, 30 s at 95°C; 30 s at 60°C, and 1 min at 72°C) with one unlabeled and one FAM-labeled primer. The final PCR fragments amplified were separated by capillary electrophoresis on a 96-capillary ABI-Prism^®^ 3730XL Genetic Analyzer (Applied Biosystems/Hitachi, Carlsbad, CA, USA). Peak area and height were measured using GeneMapper^TM^ v3.0 analysis software (Applied Biosystems). Ratios of individual peaks relative to the sum of all peaks were calculated, resulting in the relative abundance of mRNAs of the genes of interest.

### Western blot

Whole cell protein extracts were obtained by lysing 2–5 × 10^6^ cells with Laemmli sample buffer. Protein concentration was measured with the Micro BCA^TM^ Protein Assay Reagent kit (Thermo Scientific Pierce, Rockford, IL, USA). Protein extracts (25 μg) were subjected to reducing conditions before being subjected to electrophoresis on a polyacrylamide gel and then transferred to Immobilon-P membranes (Millipore, Billerica, MA, USA). One hour after blocking with 5% (w/v) non-fat milk in Tris-buffered saline with 0.1% Tween^®^-20, the membranes were incubated with the specific primary antibodies against BCL-2 (clone 124, #M0887, Dako, Denmark), BIM (clone C34C5, #2933, Cell Signaling, Danvers, MA, USA), cleaved caspase 3 (#9661, Cell Signaling), ERK2 (clone 1B3B9, #05-157, Millipore, Temecula, CA, USA), NOXA (clone 114C307, #ab13654, Abcam, Cambridge, UK), MCL-1 (clone S-19, #sc-819, Santa Cruz Biotechnology, Dallas, TX, USA), PHB1 (clone H-80, #sc-28259, Santa Cruz Biotechnology), PHB2 (anti-REA, #07-234, Millipore), PARP (#9542, Cell Signaling), PUMA (#4976, Cell Signaling), β-Actin (clone AC-15, #A5441, Sigma-Aldrich) and Tubulin (clone Ab-1, #CP06, Oncogene, Darmstadt, Germany). Antibody binding was detected using a secondary antibody conjugated to horseradish peroxidase and the enhanced chemiluminescence (ECL) detection system (GE Healthcare, Amersham Place, Buckinghamshire, UK).

### Statistical analysis

Results are shown as the mean ± standard error of the mean (SEM) of values obtained in three or more independent experiments as indicated in each figure legend. Data were analyzed using SPSS^®^ Statistics v22.0 software package (IBM^®^, Armonk, NY, USA). Two-tailed paired or unpaired Student's *t* test with normal-based 95% CI was used to compare the differences between samples, as required. Differences were considered statistically significant at *p* values below 0.05 (*p < 0.05; **p < 0.01; ***p < 0.001).

## SUPPLEMENTARY MATERIALS FIGURES


