# Supplementary material for: Targeting prohibitins induces apoptosis in acute myeloid leukemia cells
Source: Oncotarget. 2016 Aug 17;7(40):64987–5000. doi: 10.18632/oncotarget.11333 (PMC5323132; doi:10.18632/oncotarget.11333)
Supplement: Supplementary file 1 [file oncotarget-07-64987-s001.pdf]

Targeting prohibitins induces apoptosis in acute myeloid leukemia cells

Supplementary Materials

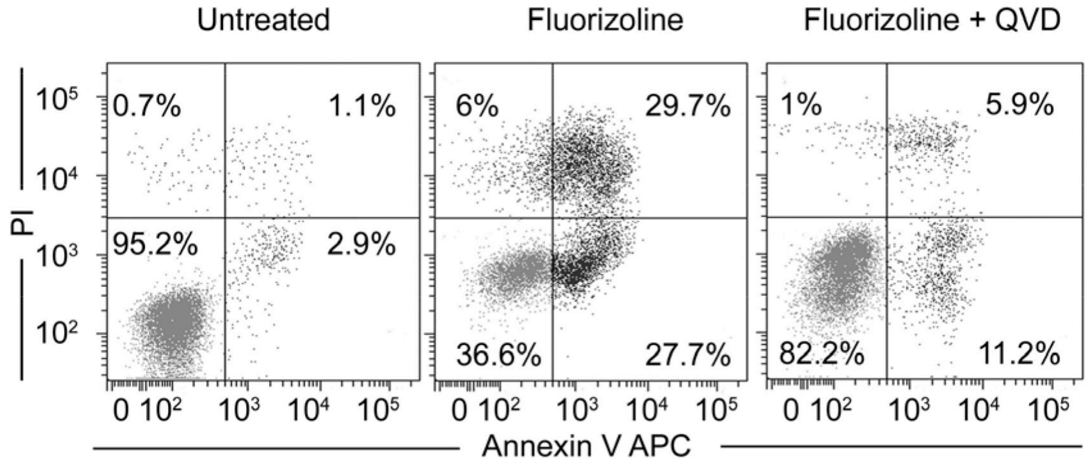

Supplementary Figure S1: Analysis of fluorizoline induced apoptosis in U-937 cells by annexin V/PI double staining. U-937 cells were untreated, treated with 10 µM fluorizoline or were pre-incubated with 20 µM caspase inhibitor Q-VD-OPh for 30 min and then treated with 10 µM fluorizoline for 24 h. Viability was measured by analysis of phosphatidylserine exposure and PI uptake. Flow cytometry plots show the percentage of each population.

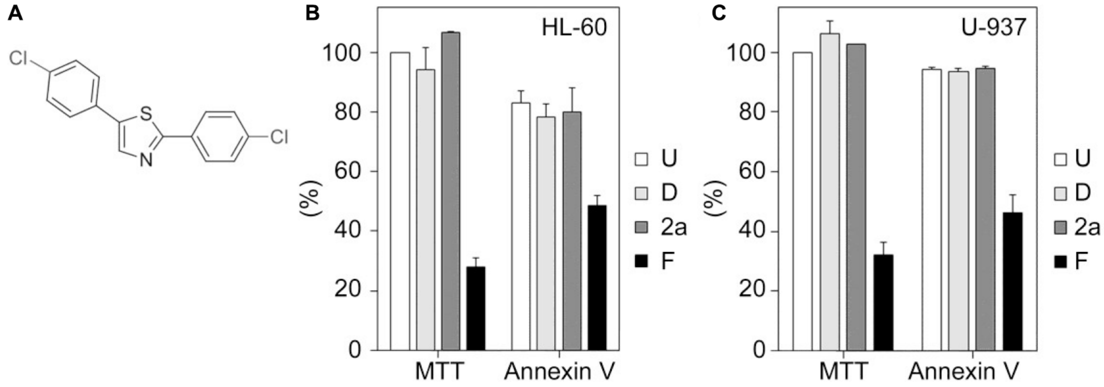

Supplementary Figure S2: The inactive analog compound 2a and the vehicle DMSO do not have cytotoxic effects in AML cell lines. (**A**) Chemical structure of the non-fluorinated diaryl thiazole synthetic precursor of fluorizoline compound 2a. (**B**) HL-60 cells and (**C**) U-937 cells were untreated (U) or incubated for 48 h with 10 µM fluorizoline (F), equivalent concentrations of DMSO (D) and 20 µM compound 2a (2a). Viability was measured by MTT metabolization (expressed as the mean ± SEM ($n \geq 3$) of the percentage of the value of untreated cells) and by analysis of phosphatidylserine exposure (expressed as the percentage of the mean ± SEM ($n \geq 3$) of non-apoptotic (annexin V negative) cells).

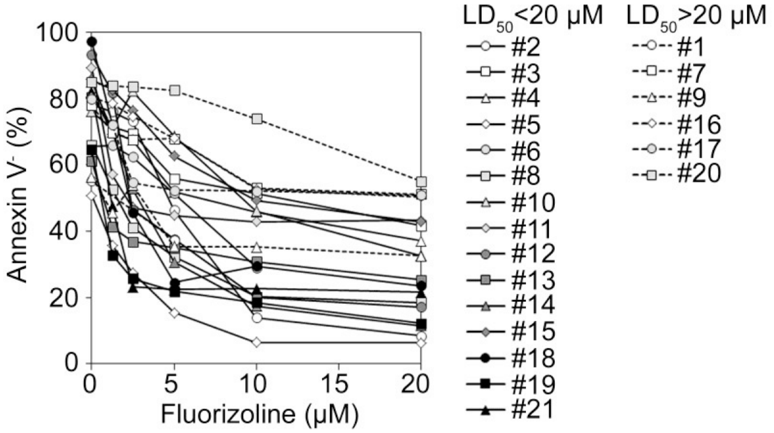

Supplementary Figure S3: Dose response of fluorizoline on primary AML cells *ex vivo*. BMMNC or PBMNC from 21 newly diagnosed AML patients were incubated for 24 h with increasing doses of fluorizoline ranging from 1.25 to 20 µM. Dashed lines represent 6 patient samples with LD_{50} values >20 µM. Viability was measured by analysis of phosphatidylserine exposure and is expressed as the percentage of non-apoptotic (annexin V negative) cells.

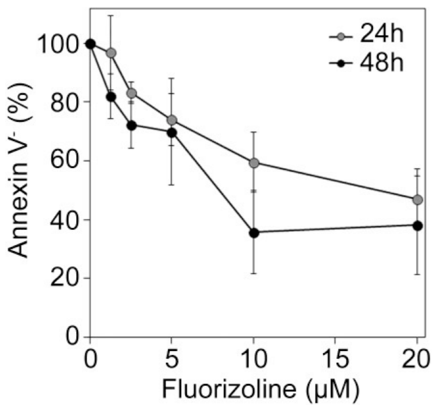

Supplementary Figure S4: Cytotoxicity of fluorizoline in relapsed primary AML samples *ex vivo*. BMMNC or PBMNC from 6 samples from AML patients after relapse were incubated for 24 or 48 h with increasing doses of fluorizoline ranging from 1.25 to 20 µM. Viability was measured by analysis of phosphatidylserine exposure and is expressed as the percentage of the viability of untreated cells. Data are shown as the mean ± SEM.

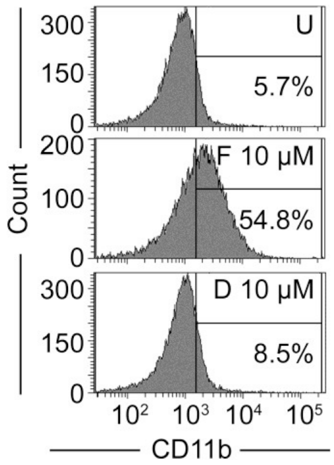

Supplementary Figure S5: Effect of fluorizoline and DMSO on the expression of the differentiation marker CD11b in U-937 cells. U-937 cells were untreated (U) or incubated for 48 h with 10 µM fluorizoline (F) or equivalent concentrations of DMSO (D). Flow cytometry histograms show the light shift of CD11b+ populations. CD11b expression was measured by flow cytometry and is expressed as the percentage of CD11b+ population.

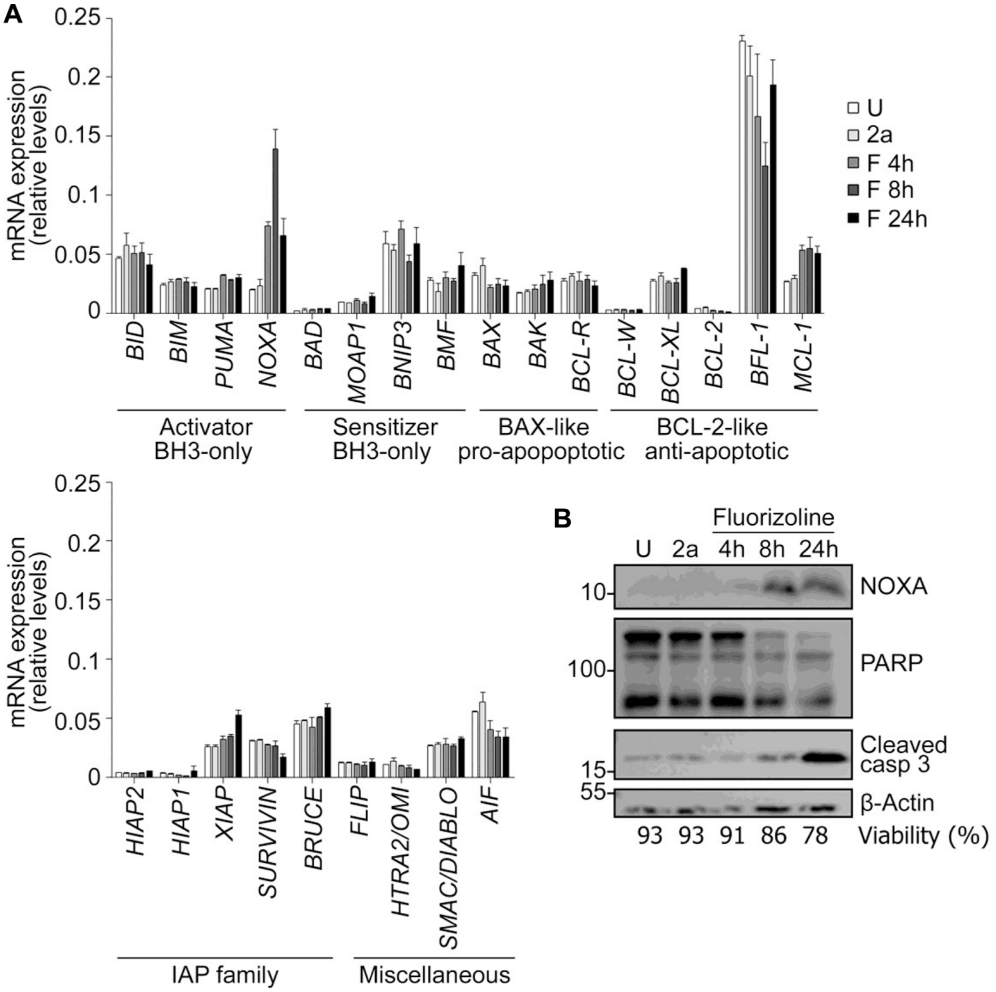

Supplementary Figure S6: Effect of fluorizoline and the structurally similar inactive analog compound 2a on the expression of apoptosis-related genes in the U-937 AML cell line. (**A**) U-937 cells were untreated (U) or treated with 20 µM compound 2a (2a) for 24 h and with 10 µM fluorizoline for 4, 8 and 24 h (F), as indicated. mRNA levels were analyzed by RT-MLPA. Data show the mean ± SEM ($n = 2$) of the mRNA expression levels. (**B**) U-937 cells were untreated (U) or treated with 20 µM compound 2a (2a) for 24 h and with 10 µM fluorizoline for 4, 8 and 24 h, as indicated. Protein levels from whole cell lysates were analyzed by western blot. β-Actin was used as a loading control. This is a representative image of two independent experiments. Viability was measured by analysis of phosphatidylserine exposure and is expressed as the percentage of non-apoptotic (annexin V negative) cells.

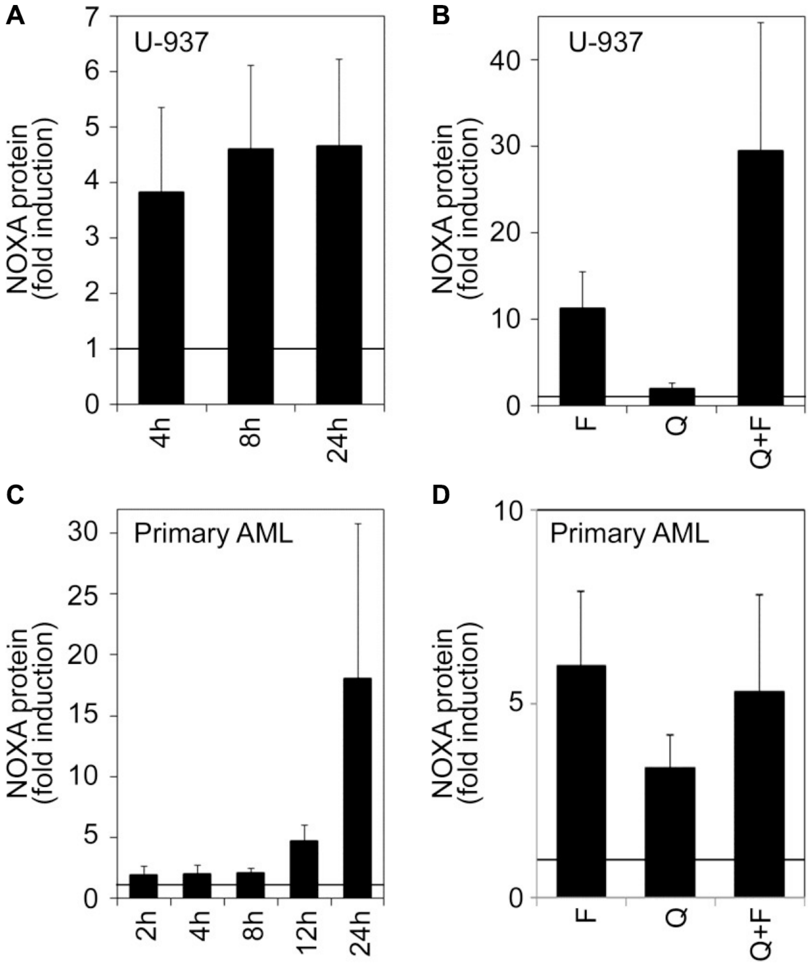

Supplementary Figure S7: Induction of NOXA protein levels upon fluorizoline treatment in AML cells. (**A**) U-937 cells were untreated or treated with 5 µM fluorizoline for 4, 8 and 24 h, as indicated. (**B**) U-937 cells were untreated or pre-incubated with 20 µM caspase inhibitor Q-VD-OPh (Q) for 30 min and then treated with 5 µM fluorizoline (F) for 24 h. (**C**) BMMNC or PBMNC from patients #13, 14, 16, 18, 20 and 21 were untreated or incubated for different times ranging from 2 to 24 h with 10 µM fluorizoline (except patient #21, whose sample was treated with 2.5 µM fluorizoline). (**D**) Cells from patients #13, 16 and 20 were pre-incubated with 20 µM caspase inhibitor Q-VD-OPh (Q) for 30 min and then treated with 10 µM fluorizoline (F) for 24 h. (A-D) NOXA protein levels from whole cell lysates were analyzed by western blot. Tubulin was used as a loading control. These values represent NOXA fold induction relative to an untreated condition. Data show the mean values ± SEM of at least three independent experiments (*n* = 4 for A; *n* = 3 for B; *n* = 4–6 for C; *n* = 3 for D).

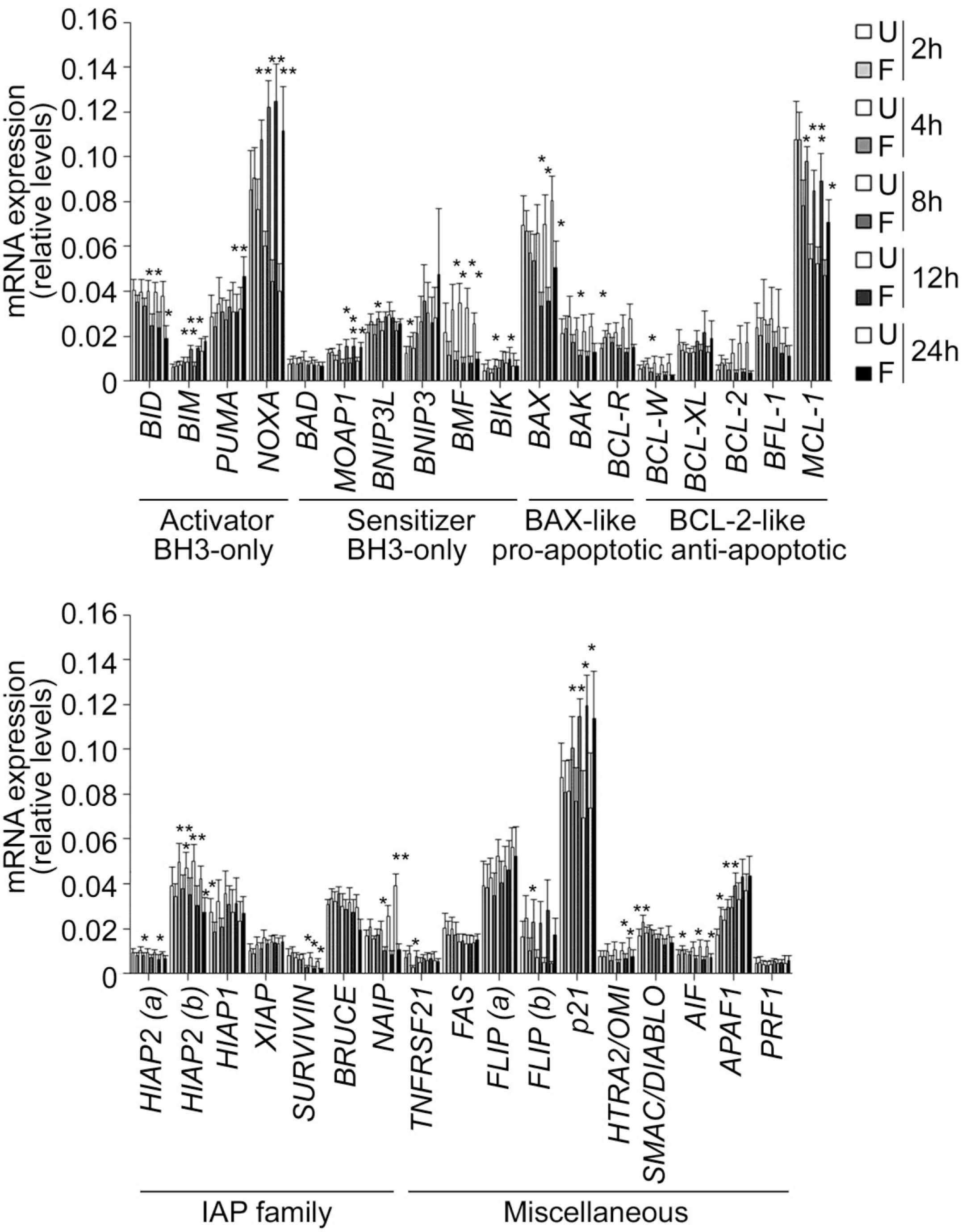

Supplementary Figure S8: Apoptosis-related gene expression profile induced by fluorizoline in primary AML samples. BMMNC or PBMNC from patients #13, 14, 16, 18, 20 and 21 were incubated for different times ranging from 2 to 24 h with 10 µM fluorizoline (except patient #21, whose sample was treated with 2.5 µM fluorizoline). RNA from cells was extracted and analyzed by RT-MLPA and the results are shown as the mean ± SEM (n = 6) of the mRNA expression levels. Two-tailed paired Student's t test significant p values are indicated: *$p \leq 0.05$, **$p \leq 0.01$, ***$p \leq 0.001$ treated *versus* untreated cells.

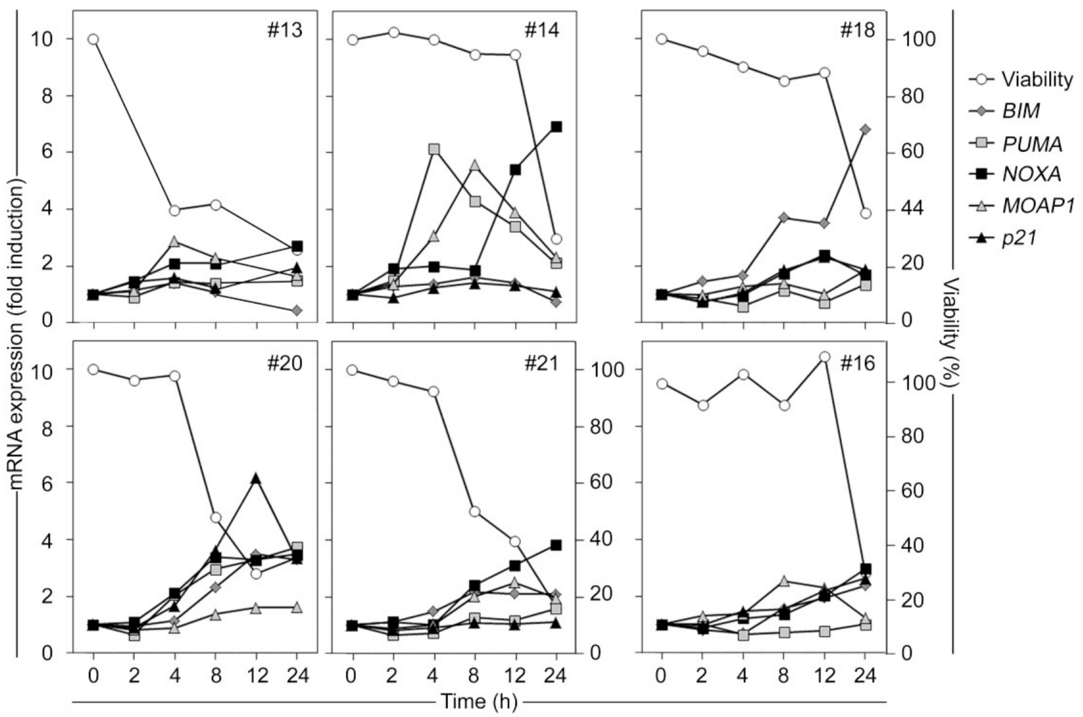

Supplementary Figure S9: Fluorizoline modulates the expression of BCL-2 family members in primary AML samples. BMMNC or PBMNC from patients #13, 14, 16, 18, 20 and 21 were incubated for different times ranging from 2 to 24 hours with 10 µM fluorizoline (except patient #21, whose sample was treated with 2.5 µM fluorizoline). RNA from cells was extracted and analyzed by RT-MLPA and the results for *BIM, PUMA, NOXA, MOAP1* and *p21* are shown as the fold induction of mRNA expression levels relative to untreated cells at each time point. Viability was measured by analysis of phosphatidylserine exposure and is expressed as the percentage of the viability (annexin V negative) of untreated cells at each time point.

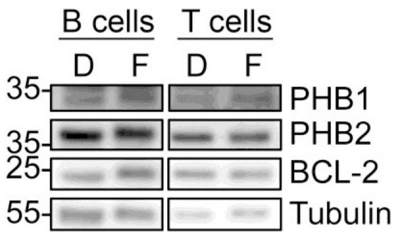

Supplementary Figure S10: Expression of PHBs in normal hematopoietic cells. Purified B cells from 2 pooled PB healthy donors and purified T cells form one individual PB healthy donor were treated with 10 µM fluorizoline (F) for 24 h or equivalent concentrations of DMSO (D). Protein levels from whole cell lysates were analyzed by western blot. Tubulin and BCL-2 were used as a loading control. This is a representative image of at least two independent experiments (*n* = 2 for B cells; *n* = 4 for T cells).
